# Evolution of SSR diversity from wild types to U.S. advanced cultivars in the Andean and Mesoamerican domestications of common bean (*Phaseolus vulgaris*)

**DOI:** 10.1371/journal.pone.0211342

**Published:** 2019-01-31

**Authors:** Tania Gioia, Giuseppina Logozzo, Stefania Marzario, Pierluigi Spagnoletti Zeuli, Paul Gepts

**Affiliations:** 1 Department of Plant Sciences/MS1, Section of Crop & Ecosystem Sciences, University of California, Davis, CA, United States of America; 2 Scuola di Scienze Agrarie, Forestali, Alimentari ed Ambientali, Università degli Studi della Basilicata, Potenza, Italy; National Cheng Kung University, TAIWAN

## Abstract

Progress in common bean breeding requires the exploitation of genetic variation among market classes, races and gene pools. The present study was conducted to determine the amount of genetic variation and the degree of relatedness among 192 selected common bean advanced cultivars using 58 simple-sequence-repeat markers (SSR) evenly distributed along the 11 linkage groups of the *Phaseolus* reference map. All the lines belonged to commercial seed type classes that are widely grown in the USA and include both dry bean and snap beans for the fresh and processing markets. Through population structure, principal components analyses, cluster analysis, and discriminant analysis of principal components (DAPC), Andean and Mesoamerican genotypes as well as most American commercial type classes could be distinguished. The genetic relationship among the commercial cultivars revealed by the SSR markers was generally in agreement with known pedigree data. The Mesoamerican cultivars were separated into three major groups—black, small white, and navy accessions clustered together in a distinct group, while great northern and pinto clustered in another group, showing mixed origin. The Andean cultivars were distributed in two different groups. The kidney market classes formed a single group, while the green bean accessions were distributed between the Andean and Mesoamerican groups, showing inter-gene pool genetic admixture. For a subset of 24 SSR markers, we compared and contrasted the genetic diversity of the commercial cultivars with those of wild and domesticated landrace accessions of common bean. An overall reduction in genetic diversity was observed in both gene pools, Andean and Mesoamerican, from wild to landraces to advanced cultivars. The limited diversity in the commercial cultivars suggests that an important goal of bean breeding programs should be to broaden the cultivated gene pool, particularly the genetic diversity of specific commercial classes, using the genetic variability present in common bean landraces.

## 1. Introduction

Common bean (*Phaseolus vulgaris* L.) is a crop plant cultivated worldwide and one of the most important grain legumes in terms of total yield and cultivated area (Food and Agriculture Organization of the United Nations (FAO) http://www.fao.org). It is grown for its green pods and immature and/or dry seeds and is a primary source of protein in the human diet in both developing and developed countries. According to FAO data (http://www.fao.org/faostat/en/#home), common bean world production in 2016 was estimated at 27 million metric t for dry bean production, and at 23.5 million t for green bean. In the United States, dry bean is grown on about 630,743 ha with a total annual production of 1,269,916 t and an average yield of 2.01 t ha^-1^, while green bean is grown on about 11,330 ha with a total annual production of 41,640 t and an average yield of 3.67 t ha^-1^ (http://www.fao.org/faostat/en/#home).

Domestication of *P*. *vulgaris* took place twice, once in the Mesoamerican area and once in the southern Andes [1], following long-distance dispersal and divergence of wild ancestral beans in the Mesoamerican area and the southern Andes [2,3]. As a consequence, two highly differentiated domesticated gene pools were established that are characterized by geographical and partial reproductive barriers (for a review, see [4]). In common bean collections, these two gene pools are strongly differentiated both for morphological and biochemical characters [5] and by different kinds of molecular markers [1,6–9]. Compared to the Andean gene pool, the Mesoamerican gene pool is characterized by either small (<25 g 100 seed weight^-1^) or medium (25–40 g 100 seed weight^-1^) seeds, with phaseolin patterns as either the “S” or “B” types; these characteristics are different from those of the of the large seeds (>40 g 100 seed weight^-1^) of the Andean gene pool with “T”, “C”, “H” and “A” phaseolin types [10,11]. Moreover, the Mesoamerican gene pool has greater genetic diversity compared to the Andean gene pool [12,13].

Following domestication, evolution within each of the two major domesticated gene pools resulted in several distinct eco-geographic races: Durango, Jalisco, Mesoamerica, and Guatemala in the Mesoamerican gene pool; and Chile, Nueva Granada, and Peru in the Andean gene pool [5,14]. Each race has distinctive physiological, agronomic, biochemical and molecular characteristics and differs from other races for allelic frequencies at specific isoenzyme or microsatellite loci [5,15,16].

In the USA, common bean varieties are classified according to market classes that are mostly characterized by distinctive seed traits (size, color, and pattern) and plant type, which are controlled by many genes [17]. USA breeders focus on dry bean classes and snap beans for the fresh and processing market both from the Andean and the Mesoamerican races. In the Andean gene pool, race Nueva Granada includes large-seeded light and dark red kidney, white kidney, bush cranberry, most green beans, and yellow beans (Mayocoba and Canario). Race Chile includes the vine cranberry beans and an array of types unique to Chile (Coscorrón and Tórtola). Race Peru is not well represented outside the center of origin, especially at higher latitudes. Within the Mesoamerican gene pool, race Mesoamerica includes the small-seeded black, white and navy beans; race Jalisco includes the Flor de Mayo and Flor de Junio beans (usually not represented outside the Mesoamerican area). Race Durango includes the medium-seeded pinto, great northern, small red, and pink beans. A fourth race unique to Guatemala includes only climbing beans types and is also rare outside the tropics. Presently, pinto is by far the most predominant market class in the USA, whereas other market classes are grown more commonly in specific production region [18]. To maintain the established market class phenotype, common bean breeders have traditionally developed new cultivars from crosses within each market class, thus, narrowing the genetic base for common bean improvement as evidenced by the low sequence variability within each market class [6,19,20]. The choice of the parents to be used in breeding programs is based first on the degree of relationships among elite materials and second on the available diversity within landraces. Common bean landraces are resources for contemporary agriculture to increase the genetic diversity of modern cultivated varieties and to meet current and new challenges of changing climate and changing market demands.

Knowledge of germplasm diversity and estimates of inter- and intra-market class genetic diversity can help common bean breeders to define new selection strategies and develop new cultivars with a broader genetic base [21]. Although several types of markers have been used in the last few decades for genetic studies, molecular markers based on microsatellite repeats (SSR—simple sequence repeat) have been used frequently in common bean because of their abundant and fairly evenly distribution in the genome, their codominant inheritance, their high levels of polymorphism and reproducibility, and their simplicity of analysis and comparisons between studies and germplasm sets. SSR markers have been confirmed as an efficient genetic tool for assessing the genetic diversity and subpopulation structure in common bean germplasm collections [22–25].

In this study, genotyping data for 58 SSR loci distributed over all 11 chromosomes were obtained to investigate the genetic structure of a collection of 192 common bean commercial USA advanced cultivars released over the last ca. 50 years and grown widely in North America. The objectives of this study were to: 1) assess the amount of genetic diversity and to describe the genetic structure of the collection; and 2) compare the analysis of modern advanced cultivars described here with the previous analysis of wild types and domesticated landraces described by Kwak and Gepts [7] for a subset of 24 SSRs. The results of this study describe the level of SSR diversity from wild types to advanced cultivars in the Andean and Mesoamerican domestications of common bean. Understanding genetic variation and relationships within and among varieties and landraces, and relationships between the many traditional races of common bean are all critical for fundamental research, conservation, and potential utilization of these genetic resources for common bean breeding.

## 2. Materials and methods

### 2.1. Plant material

A total of 192 common bean advanced cultivars representing the most popular commercial industrial seed types classes in the USA and released in the last ca. 50 years were included in this study. These genotypes were obtained from 20 American public and private breeding programs and have all been extensively used by breeders and geneticists in the USA and around the world. More specifically, the sample included 21 black, 29 navy and two small-white cultivars with small seeds (<25 g 100 seed weight^-1^), belonging to race Mesoamerica; 61 pinto and 27 great northern cultivars with medium -seeds (25–40 g 100 seed weight^-1^), belonging to the race Durango; 18 light red kidney, 11 dark red kidney, 3 white kidney, and 20 green beans with large-seeds (>40 g 100 seed weight^-1^), belonging to the Andean race Nueva Granada.

As standard genotypes, three common bean accessions were also included in the analysis: Midas, a typical Andean green bean type; BAT93, a typical Mesoamerican breeding line with multiple disease resistance [(Veranic 2 x Tlalnepantla 64) x (Jamapa x Tara)], which is also the source of the Mesoamerican genome reference sequence [2]; and Jalo EEP558, a typical Andean cultivar, which was released by a breeding program in Brazil (EPAMIG, Patos de Minas, Minas Gerais). BAT93 and Jalo EEP558 were also used as parents to obtain a recombinant inbred population that was used to develop a consensus molecular linkage map of common bean [26], in which over 170 SSR markers have been mapped [27]. A complete list of the lines, including information on pedigree, market class, and/or origin can be found in S1 Table (also freely available online at UC Davis Library Dash database at https://doi.org/10.25338/B8G30V).

Seeds were grown in a greenhouse at the University of California in Davis. After 15 days, the primary leaves were harvested and stored overnight at– 80°C.

### 2.2. Genomic DNA extraction, PCR and SSR genotyping

The frozen leaf tissue samples were lyophilized for around 48 hours using VirTis Sentry 2.0. and ground to a fine powder. Genomic DNA was then extracted from the young leaf tissue using the Qiagen DNeasy Plant Kit (Qiagen, Valencia, CA), and following the protocols provided by the manufacturer. DNA was quantified with a DyNA Quant 200 fluorometer (Hoefer Pharmacia Biotech, San Francisco, CA) and diluted to a concentration of approximately 10 ng μL^–1^ for polymerase chain reaction (PCR) amplification. The amount of genetic diversity in the 192 samples was then assessed with molecular marker analysis. A set of 58 SSR markers were selected based on their wide distribution over the *Phaseolus* genome and their high polymorphism information content (PIC) values [27–29]. More information about the SSR loci used, including the primer pair sequences, the repeat motif, and the chromosomal locations, can be found in S2 Table.

The SSRs analysis was conducted using an economical fluorescent tagging method described by Schuelke [30] in which an M13 reverse sequence tail (TGTAAAACGACGGCCAGTATGC) was added to the 5’ end of each forward SSR primers. The fluorescent dyes, 6-FAM, PET, and VIC, were attached to the 5’ end of the complementary (TGTAAAACGACGGCCAGT) M-13 universal primer sequence. For amplification, PCR reaction consisted of about 30 ng of genomic DNA, 200 μM dNTP (New England Biolabs), 0.04 μM forward primer with M-13 universal sequence tail, 0.16 μM reverse primer, 0.16 μM M-13 labeled fluorescent dye (Sigma Life Science), one unit of standard ThermoPol (Taq) reaction buffer with 2 mM MgSO4, and one unit of Taq polymerase (New England Biolabs). The PCR program consisted of 5 minutes at 94°C, 30 cycles of 30 seconds at 94°C, 45 seconds at 56°C, and 45 seconds at 72°C followed by 8 cycles of 30 seconds at 94°C, 45 seconds at 53°C, and 45 seconds at 72°C with a 10 minutes final extension period at 72°C. After dilution to a standard concentration, the amplified DNA samples were separated and sized in multiplex fashion depending on their expected size variation and were analyzed on Applied Biosystems 3730 DNA automatic analyzers.

Genotypes of markers were determined using the GeneMarker program (version1.95; SoftGenetics). When markers produced more than one peak, the peaks with clearly separated size ranges were scored independently as a different locus. This was noticed for three of the SSRs (PVag004, BM188, and BMd1). For each SSR locus and for the whole set of accessions, the total number of alleles observed were recorded and reported in a data set. Missing data were recorded when there was no detectable peak in the target size region for the marker. The data set was converted into Powermarker [31] and STRUCTURE [32] input format using the program Convert [33]. Marker data are available in S1 Table and online from the UC Davis Library database at https://doi.org/10.25338/B8G30V.

### 2.3. Statistical analysis

Based on SSRs profiles defined among the 192 studied advanced cultivars, the total number of observed alleles (N_o_), the observed (H_o_) and the expected (H_e_) heterozygosity values and PIC values were calculated across the total sample using Powermarker software version 3.25 [31]. The total number of alleles (N_a_), the mean effective number of alleles per locus (N_e_), the total number of private alleles (N_pa_), the Shannon's information index (I), the observed (H_o_) and the expected (H_e_) heterozygosity were also calculated for each different market classes as defined according to the pedigree data using the GenAlEx 6 software [34]. As the number of alleles observed is highly dependent on the sample size, the allelic richness (RS, [35]) was computed using the HP-RARE package [36], a methodology to estimate the number of alleles independent of sample size. To minimize the effects of sampling error, the number of private alleles was additionally calculated using a threshold frequency of 5% [37].

The genetic relationship among all accessions was first analyzed in two dimensions by principal coordinates analysis based on an individual-by-individual (N x N) genetic distance matrix using the program GenAlEx 6 [34] and plotted through JMP program (version 8, SAS Institute, Carry, NC). The minimum number of markers necessary to uniquely distinguishes all of the common bean advanced cultivars at one or more loci was selected using MinimalMarker software [38].

Using the model-based (Bayesian clustering) method implemented in STRUCTURE 2.3.1 software [32], the degree of population substructure was investigated without a priori information other than genotype data. A continuous series of K subgroups were tested from two to ten in twenty independents runs. The admixture model was adopted with each simulation set to a 5,000 burn-in period and 50,000 Markov chain Monte Carlo (MCMC) repetitions. To determine the optimal number of clusters [39], STRUCTURE HARVESTER [40], accessible at http://taylor0.biology.ucla.edu/struct_harvest/, was used to calculate the Delta K statistical test together with the likelihoods (posterior probabilities) of each K. To assign accessions to each different population, results from simulations with the highest likelihood among each K simulations were chosen. Potential hybrids accessions were identified if the population membership coefficient was less than 0.7. All population memberships for K = 2, K = 3, K = 5 and K = 8 are reported in S3 Table. DISTRUCT program was used to generate a STRUCTURE graphical bar plot of membership coefficients [41].

Genetic distance between accessions were calculated using the Chord distance [42]. This measure was calculated using Powermarker version 3.25 [31]. A neighbor-joining tree, using the Chord distance matrix, was then obtained using the MEGA3 software [43].

To estimate the divergence between the different population, pairwise *F*_*st*_ measurements were calculated according to Weir and Cockerham [44] using GenAlEx 6 [34]. Analysis of molecular variance (AMOVA) was also performed to assess the genetic structure of the common bean advanced cultivars using GenAlEx 6 [34]. AMOVA allowed the partition of the total SSR variation into within and among group variation components, and gave measures of intergroup genetic distance as the proportion of the total SSR variation exist in between any two groups (Phi statistics; [45]). Two levels of genetic partition were examined: (a) eco-geographic race (Nueva Granada, Mesoamerica, Durango), (b) market class subdivisions as inferred by pedigree. The significance of the resulting variance components was tested with 10,000 random permutations.

To further confirm cluster analysis and genetic structure inferred from Bayesian clustering, a discriminant analysis of principal components (DAPC), using the R package ADEGENET v2.0.0 [46] was conducted. According to this method, genetic data are first transformed using Principal Component Analysis (PCA) into components explaining most of the genetic variation. These components are then used to perform a linear Discriminant Analysis (DA), which provides variables describing genetic groups, minimizing the genetic variance within populations, while maximizing among-population variation. Further, to examine the variable allelic contributions (or ‘loading’) of each SSR locus, the ‘loadingplot’ command was used.

### 2.4. Data analysis for comparison of genetic diversity

In addition, we used a dataset published in Kwak and Gepts [7] in order to compare the 192 common bean advanced cultivars with a worldwide germplasm collection and to describe the position of the collection in the background of common bean domestication history. Deciphering the genetic structure of common bean germplasm is indeed essential for an efficient utilization of common bean diversity in breeding schemes. This dataset consisted of 349 accessions of wild and domesticated common bean [7] from the Andean and Mesoamerican gene pools (S4 Table). A subset of 24 SSR was used to compare common bean genetic diversity and correspondence between the alleles from the two different sets was carefully checked. The analyses described for the commercial lines accessions in the previous paragraphs were repeated using the integrated matrix. Relative loss of diversity in terms of genetic diversity (ΔH_e_) and alleles (ΔR_s_) was calculated according to Vigouroux et al. [47]. Wilcoxon’s signed-rank test was implemented to detect significant differences between populations on the gene diversity estimates using the software StatistiXL (http://www.statistixl.com).

## 3. Results

### 3.1. Overall SSRs diversity and polymorphism

In this study, 58 SSR markers distributed on the 11 genetic linkage groups were genotyped in 192 common bean advanced cultivars. Markers BMd-1, PVag004, and BM188 produced two very clear peaks in the expected sizes over all the samples. Therefore, they were scored as a multi-locus marker, BMd-1a, PVag004a, and BM188a and BMd-1b, PVag004b, and BM188b. According to Blair et al. [27], Yu et al. [28], and Gaitán-Solís et al. [29], BMd-1 and PVag004a were developed from an Ypr10 and Phytohemagglutinin pseudo-gene sequence, respectively, while BM188 was developed from a non-coding sequence.

The total number of alleles identified in the entire study was 343 with an average of 5.6 alleles per locus with all the SSR markers analyzed being polymorphic having from two to 22 alleles (S5 Table). The marker BM53 showed the highest number of alleles (N_o_ = 22) while the next highest allele numbers were found for BM200 (N_o_ = 20) and GATS91 (N_o_ = 18), respectively. All of them were genomic SSR markers (i.e, SSR markers developed from non-coding sequences). In contrast, the gene-based SSR markers were less polymorphic; a total of 10 markers produced only two alleles in the gene-based markers while only 3 did so in the case of the genomic markers. Hence, the average number of alleles per marker was higher for the genomic SSR (N_o_ = 7.00) when compared with the gene-based SSR (N_o_ = 3.57).

Expected heterozygosity (H_e_) of individual markers ranged from 0.046 to 0.860 (mean 0.453) for gene-based SSR and from 0.021 to 0.897 (mean 0.616) for genomic markers. The SSR markers that presented the highest expected heterozygosity (higher than 0.800) were BM53, GATS91, BM200, PV-at007, and BM187 located on linkage groups 11, 02, 01, 09, and 06 respectively. The SSR markers, which showed the lowest expected heterozygosity (lower than 0.300), were PVBR139, BMd26, BMd53, PV-atcc002, PV-atcc003, and BMd44, located on linkage groups 02, 04, 05, 07, 07, and 08, respectively (S5 Table). Observed heterozygosity (H_o_) was low, ranging from 0.000 to 0.099 and averaging 0.006 across all markers. The markers with higher observed heterozygosity were PV-at007 and BMd20 (gene-based) and PVBR107 and BM188b (genomic) (S5 Table). The observed heterozygosity could be explained by out-crossing, heterozygous genotypes, and by residual heterozygosity in breeding lines.

The PIC values, a reflection of allele diversity and frequency, were 0.496 for all the microsatellites, and ranged from a low 0.020 (BMd44) to a high 0.888 (BM53) (S5 Table).

### 3.2. Genetic diversity of the commercial types

Genetic diversity parameters were calculated for each of the races and commercial market classes (the latter based on pedigree data) using both the gene based and the genomic SSR markers (Table 1). Overall, race Nueva Granada showed greater genetic diversity for all the parameters calculated compared to the Mesoamerica and the Durango races (Table 1). The total number of alleles (N_a_) for each commercial market class varied between 1.28 (small white) and 3.30 (pinto). Dark red kidney, light red kidney, green bean, navy, black and great northern showed a very similar total number of alleles, while white kidney and small white showed a lower total number of alleles compare to the other market classes (Wilcoxon signed-rank test, P < 0.001). Similarly, the mean effective number of alleles per locus was lower for white kidney and small white (N_e_ = 1.28 and N_e_ = 1.27, respectively) compared to the other market classes, and was higher for green bean (N_e_ = 1.81) (Wilcoxon signed-rank test, P < 0.001). After size standardization, the sample of green beans still had the most alleles per locus (R_s_ = 1.35). The number of private alleles was computed considering all the commercial market classes and using an allele frequency threshold of 5% in order to reduce chances of confounding allele classification with sampling error [37]. Green beans had the most private alleles (24), followed by dark red kidney (9) and light red kidney (6) (Table 2). The Shannon's information index (I) ranged from 0.191 (small white) to 0.617 (green bean), while the expected heterozygosity (H_e_) varied from 0.137 (small white) to 0.343 (green bean). The observed heterozygosity (H_o_) was very similar among the commercial market classes and ranged from 0.000 (green bean) to 0.012 (black) (Wilcoxon signed-ranks test, P > 0.05).

**Table 1:** Genetic diversity estimates computed for all the 58 SSR loci considering the nine commercial market classes defined according to the pedigree data.

Groups	n	N_a_	N_e_	N_pa_	R_s_	I	H_o_	H_e_
*Race Nueva Granada*	*52*	*3*.*75*	*1*.*95*	*41*	*3*.*50*	*0*.*740*	*0*.*002*	*0*.*402*
White kidney	3	1.34	1.28	2	1.17	0.211	0.005	0.144
Dark red kidney	11	2.54	1.70	9	1.35	0.585	0.001	0.333
Light red kidney	18	2.21	1.42	6	1.24	0.406	0.003	0.232
Green bean	20	2.77	1.81	24	1.35	0.617	0.000	0.343
*Race Mesoamerica*	*52*	*3*.*25*	*1*.*91*	9	*3*.*08*	*0*.*637*	*0*.*009*	*0*.*343*
Navy	29	2.74	1.83	0	1.35	0.604	0.007	0.341
Black	21	2.51	1.67	2	1.29	0.511	0.012	0.283
Small white	2	1.28	1.27	7	1.18	0.191	0.008	0.137
*Race Durango*	*88*	*3*.*54*	*1*.*81*	4	*3*.*08*	*0*.*602*	*0*.*006*	*0*.*321*
Great Northern	27	2.49	1.69	3	1.31	0.540	0.007	0.308
Pinto	61	3.30	1.72	2	1.29	0.551	0.005	0.292
Total	192	3.51	1.89	-	-	0.660	0.005	0.355

n, number of samples; N_a_, total number of alleles; N_e_, mean effective number of alleles per locus; N_pa_, total number of private alleles; R_s_, allelic richness; I, Shannon's information index; H_o_, observed heterozygosity; H_e_, expected heterozygosity.

**Table 2:** Distribution of the 192 commercial market classes into the three SSRs clusters (K = 3) identified by STRUCTURE analysis based on 0.70 membership probability.

Commercial market classes	Total number of accessions	STRUCTURE cluster			Admixed accessions	% of total
		K1	K2	K3		
		Nueva Granada	Mesoamerica	Durango		
White kidney	3	3	-	-	-	*-*
Dark red kidney	11	10	-	1	-	*-*
Light red kidney	18	18	-	-	-	*-*
Green bean	20	16	-	-	4	20.0
Navy	29	-	28	1	-	-
Black	21	-	20	-	1	4.8
Small white	2	-	2	-	-	-
Great Northern	27	-	1	20	6	22.2
Pinto	61	-	-	58	3	4.9
*Overall*	*192*	*47*	*51*	*80*	*14*	7.3

### 3.3. Genetic relationship and population structure among commercial types

**Fig 1:**
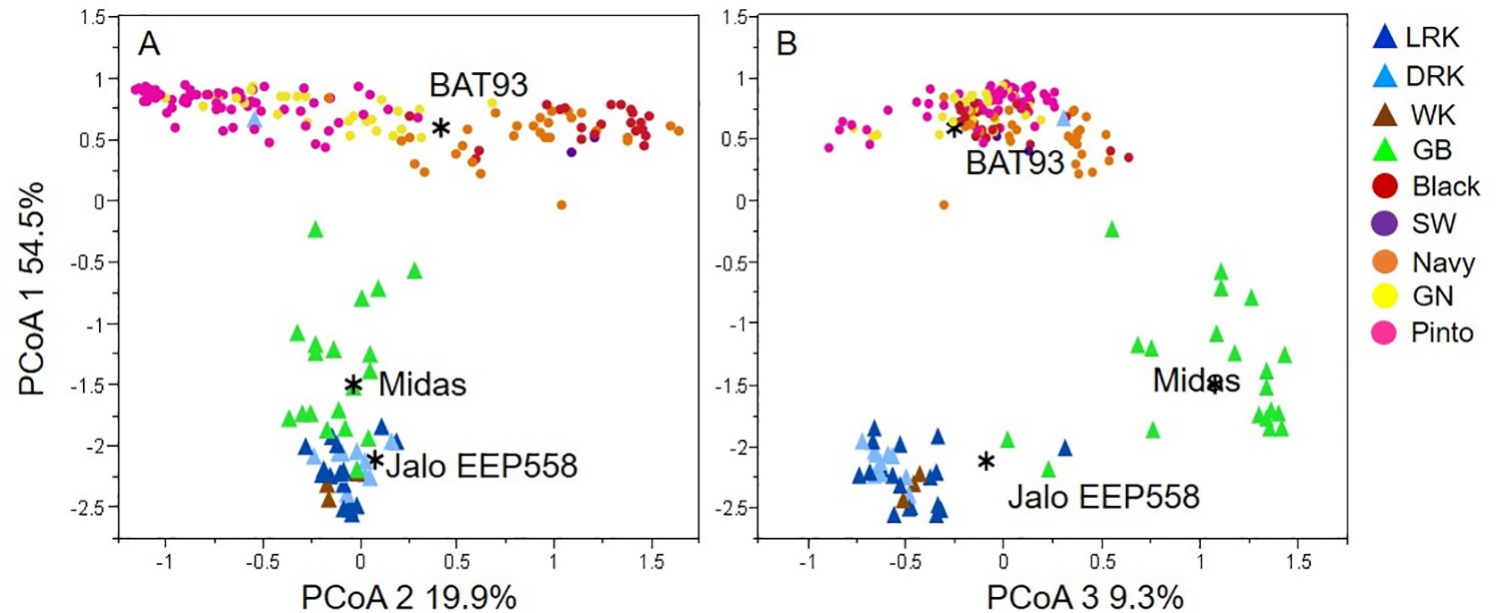
Two-dimensional principal coordinate analysis (PCoA) of SSR diversity in 192 U.S. commercial lines of common bean. A. PCoA1 vs. PCoA2. B. PCoA1 vs. PCoA3. The position of control genotypes for each gene pool is shown. The distribution of major market classes (LRK light red kidney, DRK dark red kidney, WK white kidney, GB green bean, Black, SW small white, Navy, GN great northern, and Pinto) based on pedigree data is also shown.

PCoA, Bayesian model-based clustering, and NJ tree based on genetic distance were used to investigate the genetic relationship among the different commercial advanced cultivars and to test for population structure. The PCoA approach based on a genetic distance matrix is shown in Fig 1. The two-dimensional graphical representation allows the evaluation of population structure and geometric distances between all the genotypes in the study. The most evident subdivision was that of the two major gene pools of common bean, the Andean and the Mesoamerican, which are clearly distinguished as two separate clusters (Fig 1A). The first three axes of the PCoA explained 83.7% of the variability for the whole collection. The first axis separated the Andean and Mesoamerican gene pools in the first axis (54.5% of variability explained); the second axis separated the Mesoamerican commercial cultivars (19.9%). The Andean commercial cultivars were separated in both the third (9.3%) and second axes (Fig 1B).

In Fig 1, different groups are color-coded according to the market classes based on pedigree data. The Mesoamerican advanced cultivars were separated into three major groups of the resulting plot. Black, small white, and navy accessions clustered together in a distinct group, while great northern and pinto clustered in another group, showing mixed origin (see below). The Andean advanced cultivars were distributed in two different groups. All the kidney market classes (light red kidney, dark red kidney, and white kidney) were mainly distributed in the lower left portion of the resulting plot, while the green bean accessions were more widely scattered and were distributed in between the Andean and Mesoamerican groups, possibly showing inter-gene pool genetic admixture.

**Fig 2:**
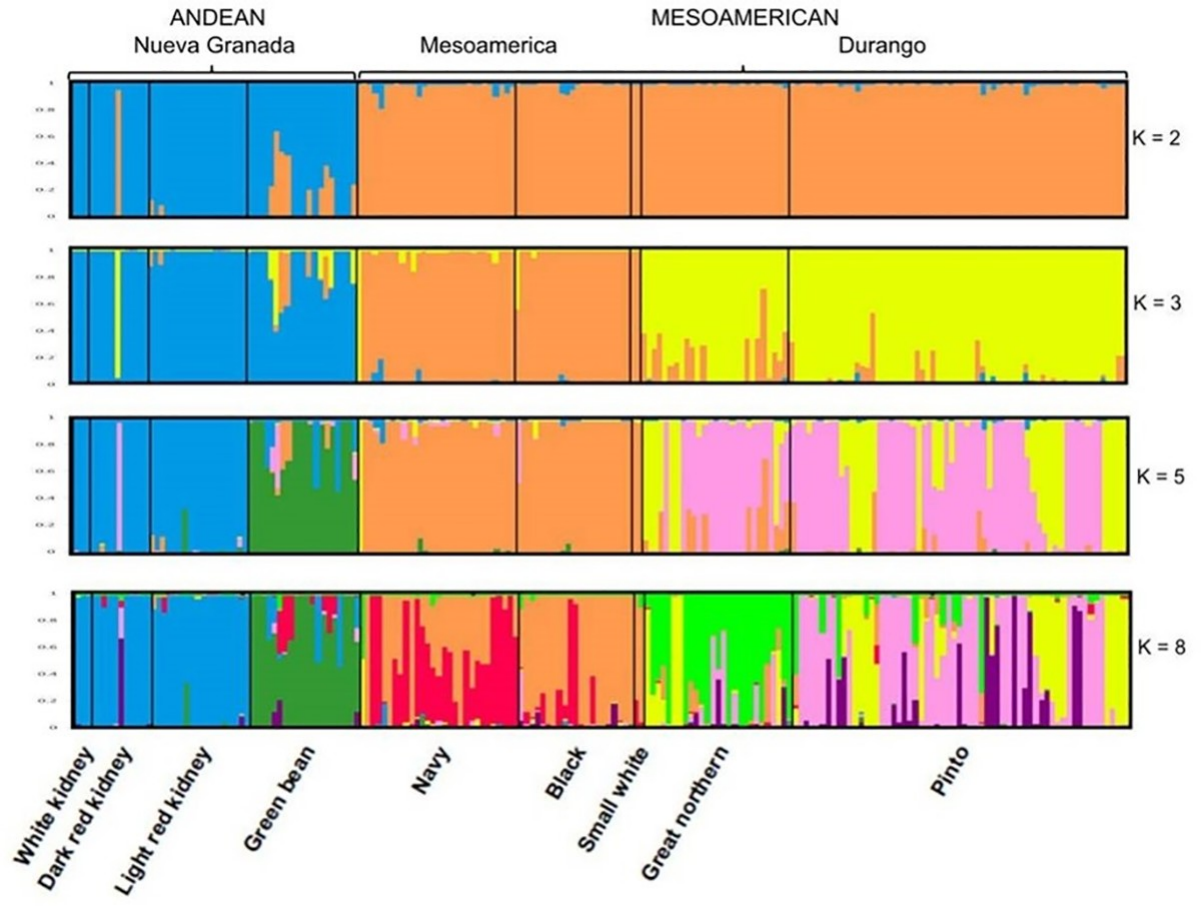
Population structure analysis for 192 advanced cultivars of Andean and Mesoamerican common bean races based on SSRs analysis. K-values of subpopulations are shown to right and naming of common bean commercial market class given below. Each individual is represented by a vertical line, and cluster assignments are indicated by color. Bar graphs were developed with the program DISTRUCT.

Population structure of the 192 commercial cultivars was further assessed using a Bayesian model-based clustering method implemented in STRUCTURE program. The number of subpopulations (K) was identified according on maximum likelihood and Delta K values [39]. The Delta K test suggested that our sample was made up of three main genetic groups (K = 3), with the next largest peak found at five clusters (K = 5) and at eight (K = 8) (S1 Fig). Separation of the subpopulations at each K value is presented in Fig 2. With K = 2, it was possible to distinguish the Andean and the Mesoamerican gene pools. Based on posterior assignment probabilities of P > 0.50, 50 accessions belonging to the kidney and the green beans market classes fell in the Andean group, while 142 accessions belonging to the pinto, great northern, navy, black and small white beans market classes were assigned to the Mesoamerican group.

For K = 3, a finer subgrouping corresponding to the three major eco-geographic races used in common bean breeding programs in the USA was obtained: the Nueva Granada race, made up of large-seeded kidney and green beans; the Mesoamerican race including the small seeded navy, black and small white; and the Durango race including the medium-seeded pinto and great northern. At K = 5, five clusters that correspond to the broad commercial seed classes that are grown in the USA were clearly separated. The first cluster (K1; blue color in Fig 2) was composed of 32 genotypes all belonging to the kidney market class (white, light and dark red kidney beans) plus one green bean. The second cluster (K2, green color in Fig 2) included 17 green bean accessions. The third cluster (K3; orange color in Fig 2) was composed by 52 accessions belonging to the small seeded navy (28), black (21), small white beans (2), and great northern (1). The fourth cluster (K4; pink color in Fig 2) included a total of 63 accessions of pinto (42), great northern (19), dark red kidney (1), and green bean (1). The fifth cluster (K5; yellow color in Fig 2) included 27 accessions belonging to the pinto class (19), great northern (7), and navy (1). An additional peak was also found at K = 8; for K = 8 an extra division was found for navy and black beans and two more division for pinto and great northern commercial market classes.

The STRUCTURE bar graphic (Fig 2) provides in addition data on the extent of admixture within the study sample. At K = 3, 178 accessions out of 192 (93%) were assigned to one or another group with more than 70% posterior probability (Table 2 and Fig 2). The remaining 14 non-assigned accessions (7%) were assumed to have a mixed ancestry. The admixture was clearly observed in the great northern class (6 accessions; 22%), green bean class (4 accessions; 20%), pinto class (11 accessions; 18%) and black class (1 accession; 5%) (Table 3). At higher K values, more accessions showed admixed ancestry. For K = 5, 28 accessions (15%) were assumed as having admixed ancestry. The admixture was greater in the green bean market class (8 accessions; 40%), great northern market class (7 accessions; 26%), and pinto market class (11 accessions; 18%). The potential green bean hybrids resulted from hybridizations between different subgroups within the Mesoamerican group and the Andean group, while the great northern and the pinto hybrids always resulted from hybridizations between different subgroups within the Mesoamerican group.

**Table 3:** Analysis of molecular variance (AMOVA) for the 192 common bean advanced cultivars for two models of genetic structure based on 58 SSR markers.

Source of variation	df	Sum of squares	Variance components	Percentage of total variance	SSR - ϕ PT	P-value
Between races Nueva Granada, Mesoamerica, Durango	2	4731.475	40.471	34	0.342	<0.0001
Between market classes within race	2	950.327	14.978	18	0.280	<0.0001
Among accessions within market class within race	187	7109.318	38.536	47	0.526	<0.0001
Total	191	12791.120	93.985			

df: degree of freedom; significance tests with 10,000 permutations.

**Fig 3:**
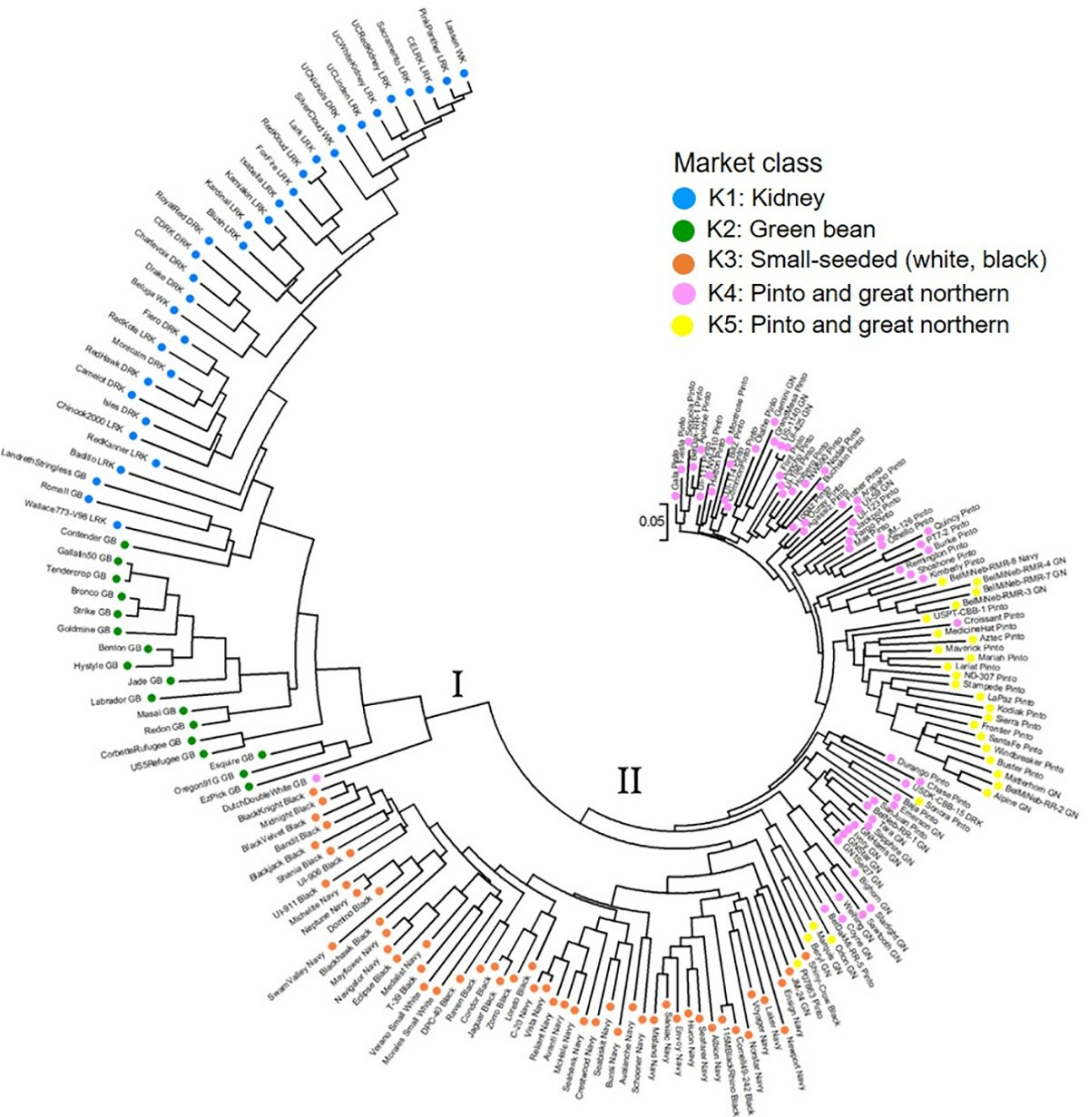
Neighbor-joining tree of SSRs diversity for the 192 advanced cultivars based on the C. S. Chord distance. Each branch is color-coded according to membership into the K = 5 groups identified by STRUCTURE (same colors as in Fig 2). K1 (Blue): Kidney market class; K2 (Green): Green bean market class; K3 (Orange): Small-seeded (white, black) market class; K4 (Pink): Pinto and great northern market classes; K5 (Yellow): Pinto and great northern market classes.

An NJ clustering tree corroborates the population assignments inferred by STRUCTURE and the PCoA (Fig 3). In order to compare the results of the phylogenetic analysis with the assignment of individuals to groups using STRUCTURE software, the branches of the tree were colored according to STRUCTURE simulations for preset K = 5 (same colors as STRUCTURE bar plot of membership coefficients for K = 5 in Fig 2). Two major cluster were detected. Cluster I consisted of 51 Andean accessions, and cluster II of 141 Mesoamerican accessions (Fig 3). This finding is consistent with the gene pool subdivision identified with STRUCTURE at K = 2, with the exception of one green bean accession (Dutch Dubbele Witte line), which grouped with the Andean accessions but was considered a Mesoamerican accession by the STRUCTURE analysis. For these accessions STRUCTURE coefficients indicated a membership lower than 70% in the corresponding population. Furthermore, the Andean cluster I was divided into two main sub-clusters which were equivalent to groups k1 (kidney beans) and k2 (green beans) identified by STRUCTURE analysis at K = 5 (Fig 3). The Mesoamerican cluster II was further divided into two main sub-clusters. These groups were mostly equivalent to the race Mesoamerica and race Durango (Fig 3).

The great northern advanced cultivar ‘GN#1Sel27’ and ‘GN Star’ and the green bean cultivar ‘Gallatin50’ and ‘Tendercrop’ generated the same profiles with all 58 SSR markers. The minimum marker set for discrimination of all tested common bean advanced cultivars (except for the four cultivars just mentioned) included the following 18 SSRs: Bmd10, GATS91, PVBR107, BM210, BMd41, BM188B, BMd47, PVBR54, BM53, BMd20, BMd37, PV-ctt001, BMd25, BMd28, BM171, BM200, BM114, BM141.

### 3.4. Population differentiation

AMOVAs were conducted to determinate the variation explained by races (Nueva Granada, Mesoamerica, and Durango) and market class groups (Table 3). The results indicated that 34% of the genetic variation (P < 0.0001) resided among races and 18% (P < 0.0001) resided between market classes within-race. The remaining 47% of the total variation (P < 0.0001) was explained by accessions within market class within race.

Genetic variation between races and market classes was also tested using the *F*_*ST*_ statistic estimated from pairwise comparisons as a measure for genetic distance between races and market classes. Within each race, the degree of genetic differentiation was very large, typical of inbreeding species (range: *F*_*ST*_ = 0.529–0.291; S6A Table). All pairwise comparisons among the market classes were significant (S6B Table). The degree of differentiation between any market classes was moderate to very large (range of *F*_*ST*_ = 0.061–0.738). In general, *F*_*ST*_ values between subgroups within gene pools were generally low compared to *F*_*ST*_ values of subgroups between gene pools. Low genetic differentiation was found between pinto and great northern groups and between navy and black beans. Moreover, in the Andean gene pool green beans were less differentiated compared to the kidney groups and closer to the Mesoamerican gene pool.

### 3.5. Cluster analysis using DAPC and validation of STRUCTURE results

**Fig 4:**
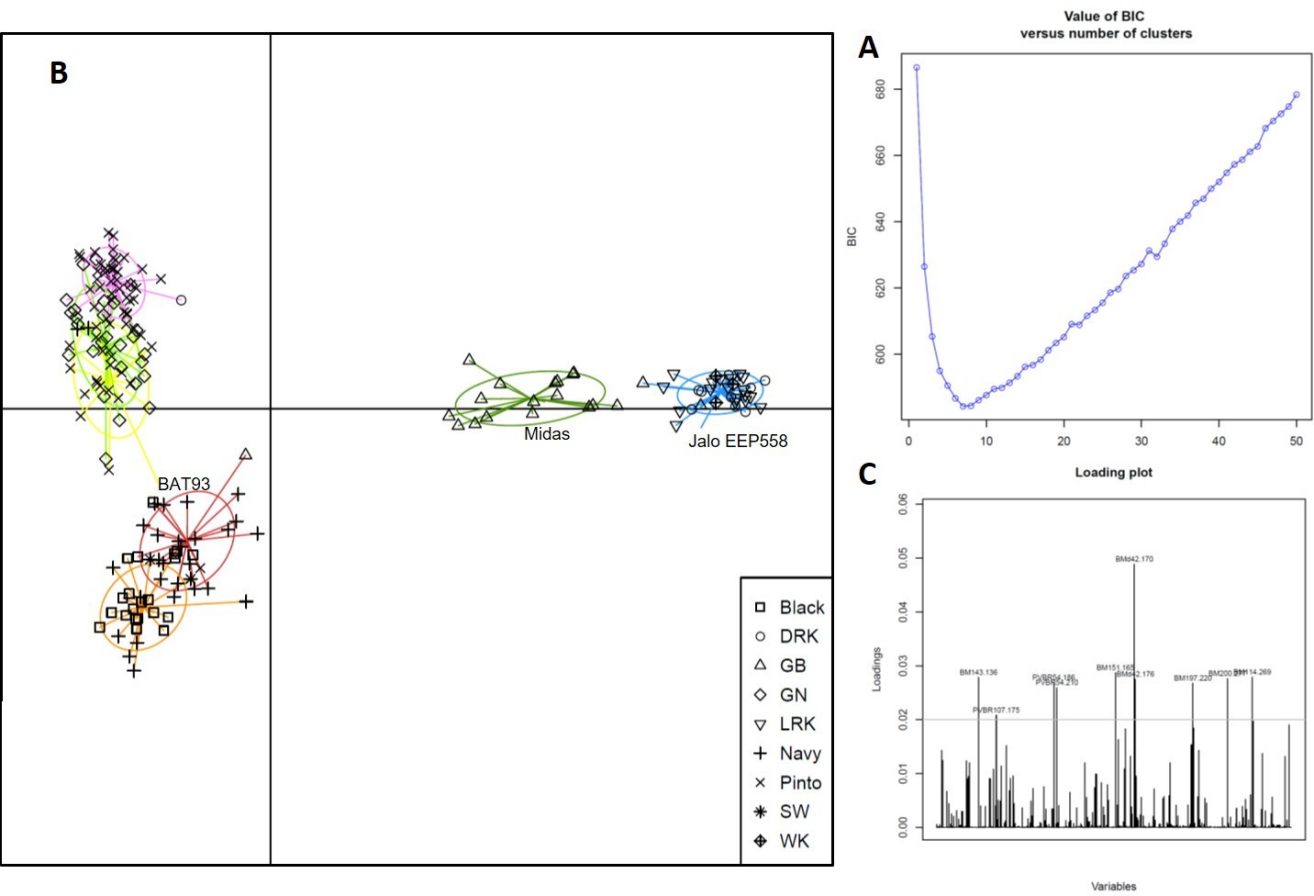
Results of DAPC applied to the 192 commercial advanced cultivars of common bean. A. Bayesian Information Criteria (BIC) for increasing values of the number of clusters. The chosen number of clusters was K = 7. B. Scatterplot of the first two principal components of the DAPC on the common bean collection. Individuals are represented by symbols according to their market class. Colours and inertia ellipses identify the clusters. C. Loading plot generated from using all 58 SSR, with the horizontal line representing an arbitrary threshold value of .002. Illustrates which SSR contributed most to the individual principle component analysis.

We validated maximum likelihood-based clustering results from STRUCTURE analysis using the Discriminant Analysis of Principal Components (DAPC) method, which is considered free of Hardy-Weinberg and linkage disequilibrium assumptions [48]. Model selection using the Bayesian Information Criterion (BIC) revealed the presence of hierarchical structure in the population, with a steep decline from K = 2 up to around K = 6 followed by a gentler decrease. The lowest BIC value, which corresponded to an optimal cluster number, was obtained at K = 7 (Fig 4A). DAPC clustering recapitulated the groupings uncovered by both the distance-based hierarchical clustering topology as well as ancestry estimates achieved by STRUCTURE (Fig 4B). Comparison of the cluster membership results from the DAPC and STRUCTURE analyses are summarized in Fig 4B. A major difference between the results of the two clustering methods was the propensity of the DAPC analysis to assign individuals to a single cluster compared to STRUCTURE, which was able to assign admixed individuals to multiple clusters. One interesting feature of the DAPC method is that it allows calculating the contributions of alleles to the regions of the genome driving genetic divergence among groups [48]. Alleles of markers PVBR107, PVBR54, BM143, BM197, BM151, BM114 and BMd42 (on LG2, LG3, LG8, LG9, LG10) contributed most to the individual principal component analysis (Fig 4C).

### 3.6. Estimation of the relative loss of diversity due to domestication and breeding

For a subset of 24 SSR markers, we compared and contrasted the genetic diversity data obtained in the present study with those obtained in another study that used wild and domesticated landrace accessions of common bean [7]. To allow direct comparisons of the domesticated landraces and advanced cultivars, we reanalyzed the data from Kwak and Gepts [7] using the same statistics described previously in order to estimate the relative loss of diversity due to post-domestication and breeding process and to understand how much genetic diversity is still available for breeding purpose. Table 4 provides an overview of genetic variation detected by SSR markers within and among the Andean and the Mesoamerican races for both domesticated landraces and modern commercial cultivars of common bean. Considering the Andean gene pool, the results obtained showed that the common bean cultivars were characterized by a lower number of alleles (N_a_ = 3.67), number of effective alleles (N_e_ = 1.88), allelic richness (R_s_ = 3.16), Shannon's information index (I = 0.73), and expected heterozygosity (H_e_ = 0.394) than the landrace samples (N_a_ = 7.79; N_e_ = 2.80; R_s_ = 5.14; I = 1.08; H_e_ = 0.474) (Wilcoxon signed-rank test, P < 0.001). For the Andean gene pool, the total reduction in diversity in terms of number of alleles (ΔN_e_), allele richness (ΔR_s_), and genetic diversity (ΔH_e_) attributable to post-domestication was 0.33, 0.39, and 0.17, respectively. This loss of diversity is significantly greater than the loss of diversity attributable to domestication (comparisons between wild and landraces, ΔN_e_ = 0.30; ΔR_s_ = 0.13; ΔH_e_ = 0.07) (Table 4).

**Table 4:** Diversity and relative diversity loss between landraces and commercial advanced cultivars of Andean and Mesoamerican common bean races.

Genepool	n	N_a_	N_e_	ΔN_e_	R_s_	ΔR_s_	I	H_e_	ΔH_e_	H_o_
***Andean***										
*Wild*	*31*	*6*.*75*	*4*.*01*		*5*.*93*		*1*.*187*	*0*.*507*		*0*.*015*
*Landraces*	*109*	*7*.*79*	*2*.*80*	*0*.*30*	*5*.*14*	*0*.*13*	*1*.*083*	*0*.*474*	*0*.*07*	*0*.*004*
Race Nueva Granada	55	4.63	1.89				0.717	0.350		0.003
Race Chile	18	4.04	2.52				0.925	0.465		0.007
Race Peru	36	5.33	2.90				1.064	0.501		0.005
*Varieties*	*52*	*3*.*67*	*1*.*88*	*0*.*33*	*3*.*16*	*0*.*39*	*0*.*730*	*0*.*394*	*0*.*17*	*0*.*000*
Race Nueva Granada	52	3.96	1.87	*0*.*01*			0.781	0.420	*-0*.*20*	0.000
**Mesoamerican**										
*Wild*	*59*	*8*.*83*	*4*.*97*		*8*.*19*		*1*.*406*	*0*.*588*		*0*.*040*
*Landraces*	*66*	*6*.*63*	*3*.*27*	*0*.*34*	*6*.*04*	*0*.*26*	*1*.*113*	*0*.*500*	*0*.*15*	*0*.*006*
Race Mesoamerica	21	4.50	2.84				0.967	0.462		0.004
Race Jalisco and Durango	45	5.38	2.68				0.948	0.444		0.006
*Varieties*	*140*	*5*.*04*	*2*.*38*	*0*.*27*	*4*.*09*	*0*.*32*	*0*.*830*	*0*.*418*	*0*.*16*	*0*.*003*
Race Mesoamerica	50	3.58	1.88	0.34			0.673	0.355	*0*.*23*	0.007
Race Durango	90	4.25	2.09	0.22			0.689	0.342	*0*.*23*	0.001
**Total**	466	6.02	3.10		–		1.024	0.473		0.014

In the Mesoamerican gene pool, the common bean advanced cultivars presented a lower number of alleles (N_a_ = 5.04), number of effective alleles (N_e_ = 2.38), allelic richness (R_s_ = 4.09), Shannon's information index (I = 0.83), and expected heterozygosity (He = 0.418) than the domesticated samples (N_a_ = 6.63; N_e_ = 3.27; R_s_ = 6.04; I = 1.11; H_e_ = 0.500) (Wilcoxon signed-rank test, P < 0.001). The total reduction in diversity in terms of number of alleles (ΔN_e_), allele richness (ΔR_s_), and genetic diversity (ΔH_e_) attributable to post-domestication for the Mesoamerican gene pool was 0.27, 0.32, and 0.16, respectively (Table 4).

Considering only the domesticated landraces and the modern advanced cultivars, we computed the number of private alleles imposing a minimum allele frequency threshold of 5% in order to reduce chances of confounding allele classification with sampling error [37]. The number of private alleles in the Andean landraces was 11, while the Andean modern advanced cultivars showed only five private alleles. In the Mesoamerican gene pool, 15 private alleles were found in the landraces and four in the advanced cultivars.

If we compare the Andean and Mesoamerican advanced cultivars, there was a clear difference in the level of genetic diversity: the Andean gene pool showed a substantially lower level of genetic diversity than the Mesoamerican gene pool for all of the genetic diversity estimates (Table 4). Compared to the Mesoamerican gene pool, there was a much higher reduction in diversity associated with the process of post-domestication and breeding in the Andes. This trend is opposite to what was observed for the domestication process, where the reduction of diversity was larger for the Mesoamerican gene pool compared to the Andean gene pool (Table 4). If we look at the race Nueva Granada we can clearly see an increase of diversity from landraces to vanities (ΔH_e_ = -0.20). This increase in diversity is because green beans where included in this group. Indeed, some green beans originated in the Mesoamerican gene pool, showing admixture origin. If we analyze the loss of genetic diversity in race Nueva Granada without including the green bean group the genetic diversity (ΔH_e_) attributable to post-domestication was 0.25.

Race Mesoamerica showed a marked reduction in genetic diversity between the landraces descendants and their respective commercial advance cultivars (ΔN_e_ = 0.34; ΔH_e_ = 0.23). The limited diversity in the commercial cultivars has serious consequences for bean breeding, and suggest that an important goal of bean breeding programs should be to broaden the cultivated gene pool.

### 3.7. Genetic structure of the common bean advanced cultivars and their relationship with the landraces

Our aim was to describe the position of common bean advanced cultivars in the background of bean domestication history. For that purpose, we first described the genetic structure of the common bean collection of wild and landrace accessions using DAPC, then we projected the common bean advanced cultivars as supplementary individuals on the obtained clustering. We chose K = 11, a value from which the BIC decreased or increased only by negligible amounts in many runs of the analysis.

**Fig 5:**
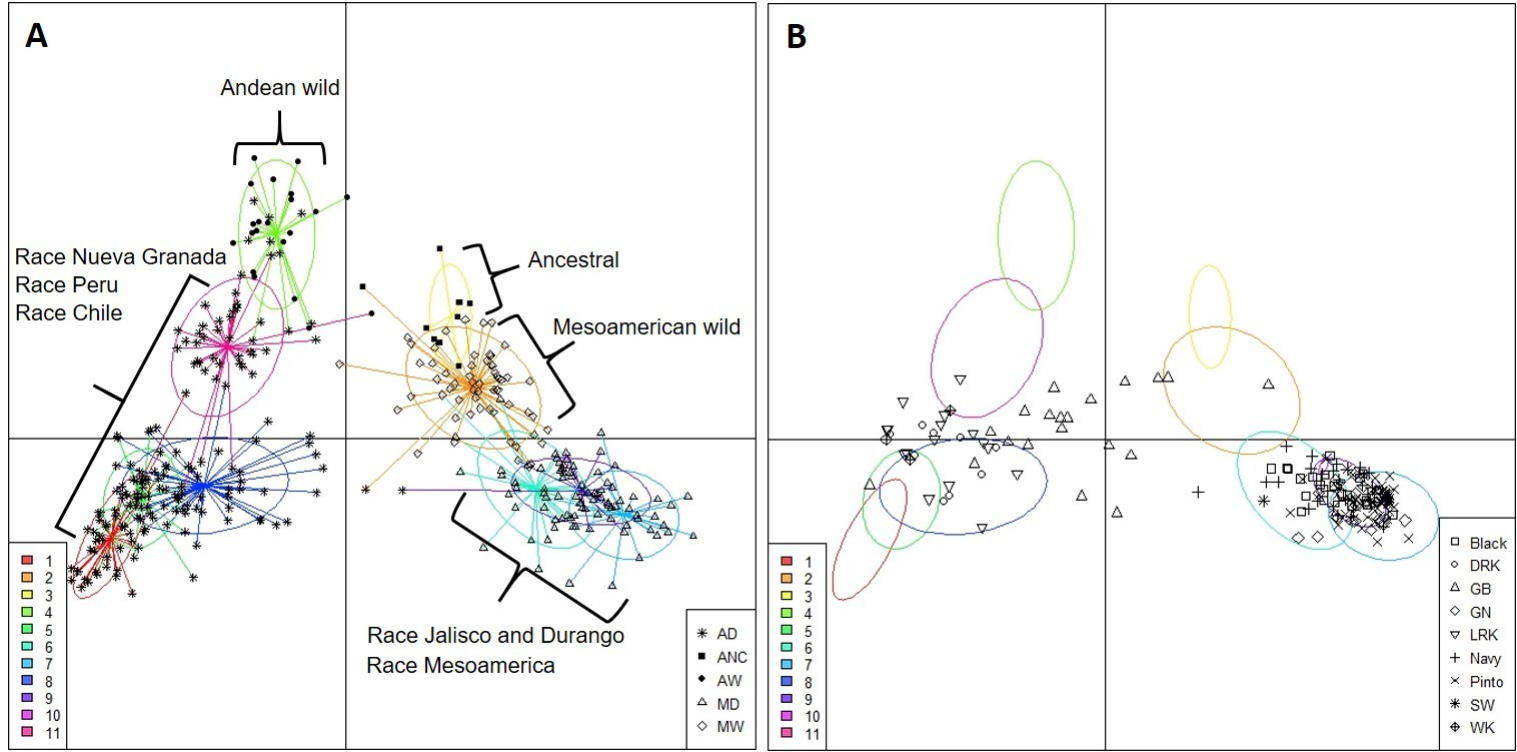
A. Results of DAPC applied to a worldwide germplasm collection of 349 wild and domesticated landraces of common beans [7]. B. Scatterplot of the projection of the 192 common bean advanced cultivars on the genetic clusters identified by DAPC on the worldwide germplasm collection. The clusters are represented by their inertia ellipses.

Among the 11 clusters identified (Fig 5A, S4 Table), four corresponded both to a homogeneous composition regarding the evolutional position and to a relatively high average membership probability: a cluster with all wild ancestral accession (C3), two cluster consisting of wild Mesoamerican individuals (C2 and C10), and a cluster made up of wild Andean accessions (C4). Three more clusters were of race Mesoamerica accessions (C6) and race Jalisco and Durango accessions (C7 and C9),. Two clusters were of the Andean races Peru and Chile (C11) and race Nueva Granada (C8). One cluster (C5) was a mixture of the three different Andean races, race Peru, Chile and Nueva Granada. An isolated cluster (C1) was found that included only accessions of race Nueva Granada. Interestingly, this homogeneous cluster made up only of race Nueva Granada accessions included almost exclusively landraces with yellow colored seeds and coming from Mexico (S4 Table).

The common bean advanced cultivars from the USA projected as supplementary individuals are showed in Fig 5B. The Mesoamerican advanced cultivars were mainly assigned to the two clusters consisting of race Mesoamerican and race Jalisco and Durango (C6 and C9). The Andean kidney market class (white, light and dark red kidney beans) were distributed in two cluster (C5 and C8), while the green bean market class was distributed in three clusters (C2, C8, C9), showing clearly an introgressed origin. Interestingly, most cultivars in the domesticated cluster C1 were the yellow-colored Canario beans.

## 4. Discussion

### 4.1. Genetic diversity in common bean advanced cultivars

Progress in common bean breeding program requires the exploitation of genetic variability among genotypes of different market classes, races and gene pools. A variety of approaches, from morphological data to molecular markers including phaseolin seed protein [10,49], RFLPs [50], RAPDs [26], AFLP [51,52], SSR [7,16,53], SNPs [54,55], and DNA sequences [2,13,56,57] have been applied for assessment of genetic variability among common bean genotypes. In the present study, we assessed usefulness of SSR markers to investigate genetic variability and relationships among 192 common bean advanced cultivars. The common bean accessions used in this study represent the diversity in the most advanced USA breeding stages of major public and private variety development programs in common bean and encompass the marketable gene pools available in the country.

Allelic diversity analysis in this study revealed that the total number of alleles amplified at 58 SSR loci in 192 commercial cultivars was 343 (5.62 alleles per locus on average, ranging from 2 to 22). Although number of alleles may not be the best statistic to compare genetic diversity of different samples, in general, our sample presented a lower number of alleles than the sample of 44 genotypes (parents of mapping populations) used in the study of Blair et al. [58], considering a shared set of 48 SSR markers. Our values were also lower than the previously reported estimates of SSR marker diversity in a common bean core collection [59]. Allele numbers ranged from 2 to 76 (mean 18) for the above-mentioned study. The low SSR allele diversity found in this sample may reflect the impact of plant breeding in common bean. These comparisons should be considered with caution, because all these values can be altered depending on both the sample size and the SSRs or other markers, selected for the analysis.

The expected heterozygosity value (H_e_) was 0.546 (0.601 for genomic SSR markers and 0.466 for gene–based markers), indicating that the common bean advanced cultivars displayed a substantial genetic diversity. Since estimates of expected heterozygosity are not affected by differences in sample size [60], direct comparison between different studies but also different accession pools are possible. Our finding was in accordance with the results of an earlier study on SSR diversity in common bean (0.527) [58]. However, while for the whole sample genetic diversity is substantial, genetic diversity within gene pool and more importantly within market classes variability is more limited. We found similarly low level of expected heterozygosity in both gene pools as well in the different market classes (values ranged from 0.137 to 0.341). This was consistent with previous studies based on sequencing experiments [6,20].

### 4.2. Population structure and genetic diversity among commercial types

As an assessment of the structure of our common bean commercial types, we used a model-based clustering method. As expected, at K = 2, STRUCTURE analysis showed a significantly different population structure between Mesoamerican and Andean accessions. The accessions split almost entirely into two distinct groups by gene pools with accessions falling into the group from their center of origin, either Mesoamerica or Andes. The results above were also confirmed by PCoA analysis. The Mesoamerican and Andean gene pools were almost completely separated by the center of the vertical axis. It has been shown that the resolution of PCoA methods and STRUCTURE are quite similar in many cases [61]. At K = 3, advanced cultivars were clearly separated into three different eco-geographic races: Mesoamerica race, including the small-seeded Navy and black beans; Durango race, with pinto and great northern beans; Nueva Granada race, including large-seeded kidney beans and most snap beans.

The genetic structure at K = 5, allowed us to distinguish the major market classes in common bean. Diaz and Blair [16] evaluated the diversity of a common bean sample using microsatellites and also observed that microsatellite diversity was correlated with commercial types, as genotypes were separated fairly accurately according to seed size and color. In our study all market classes are clearly separated in each cluster, excepted for the great northern beans, which were mixed with some pinto beans. Although these advanced cultivars can be distinguished by their plant and seed morphology, they were not well differentiated at the molecular level in this study. The closeness of the two advanced cultivars may be due to a differentiation limited to a few major genes controlling plant and seed morphology (including color and color pattern), or the advanced cultivars of this group may share a common genetic origin, potentially originating from a unique lineage extensively used as progenitor. A second observation was that the green bean cultivars we analyzed belonged to the Andean gene pool rather than to the Mesoamerican one. Furthermore, the green bean cluster was differentiated from other Andean populations on coordinate 2 (20%; Fig 1) and was positioned close to the Mesoamerican accessions along coordinate 1 (54%) in PCoA plots. Thus, unlike most other commercial classes which originate in a single gene pool (Andean or Mesoamerican), green bean accessions, as a group, may actually have been derived from both Mesoamerican and Andean germplasm. Moreover, results of private alleles using 58 SSRs indicated that the green beans (mean expected heterozygosity: 0.343; private alleles: 24; allelic richness: 1.35) actually had higher genetic diversity than the other groups. This implied there were more potentially new alleles in the green bean compared with the other market classes due to introgression between the two gene pools.

To provide an additional, and rather different, type of algorithm against which to compare our structure, we also analyzed the data using genetic distance for each pair of genotypes. In our study, the C.S. Chord distance [42] was selected to calculate the genetic distances between individual samples because it does not require a specific mutation model to account for microsatellite evolution [62]. Neighbor-joining tree construction allowed a clear separation between advanced cultivars from Mesoamerican and Andean South American domestication centers. These results are in accordance with the STRUCTURE, PCoA and AMOVA analysis.

Significant fixation index *F*_*ST*_ also revealed genetic substructure within the common bean advanced cultivars with the pinto-great northern beans and navy and black beans being the less different from the other varietal classes. The lower genetic differentiation between pinto and great northern groups and between navy and black beans is consistent with their membership in races Durango and Mesoamerica, respectively, and may indicate a shared ancestry pedigree. Moreover, in the Andean gene pool green beans were less differentiated compared to the kidney groups and closer to the Mesoamerican gene pool, which may indicate multiple origins of green beans in the Andean and Mesoamerican gene pools or introgression between these gene pools.

### 4.3. Overall reduction in genetic diversity in Andean and Mesoamerican gene pools, from wild to landraces to advanced varieties

We focused on the comparison between the domesticated landraces and the advanced cultivars, to identify and understand possible changes that have occurred during the post-domestication and breeding process in common bean and to better use the genetic diversity in breeding programs and germplasm collections. An overall reduction in genetic diversity in the Andean and Mesoamerican gene pools, from wild to landraces to advanced varieties was observed. This reduction is somewhat tempered by the fact that the markers used—SSRs—are very variable and that some of the loss of diversity has been compensated by mutations since the original dispersal, selection, and breeding. This makes it look like both domestication and current breeding have a similar effect on diversity, whereas other, less variable markers may show a stronger effect of domestication. A previous analysis by Sonnante et al. [19], using M13-related RFLP markers, also identified a progressive decrease of genetic diversity from wild beans to landraces to improved varieties: compared to wild beans and landraces, cultivars in the USA were devoid of genetic diversity for this type of marker.

### 4.4. Cluster analysis using DAPC

Based on DAPC analysis the common bean collection made of wild, landraces and advanced cultivars could be divided into 11 subgroups. The DAPC analysis was a useful tool to investigate the population structure of the common bean collection. The clustering of the advanced cultivar presented in this study may give interesting cues for increasing diversity in breeding programs and germplasm collections. For instance, advanced cultivars were included in all domesticated clusters except for cluster C1, which included Mexican landraces of race Nueva Granada with yellow seed. Also, most Mesoamerican advanced cultivars were included in one cluster. These findings are in agreement with the repeated use of a few founder genotypes that played a relevant role in the creation of the genetic basis of modern genetic pools, becoming in turn progenitors of new elite varieties and completely replacing traditional landraces. The continued use of these genotypes made the gene pool smaller for all of the common bean commercial cultivars and resulted in the loss of genetic diversity. Therefore, new variability should be incorporated into the existing elite germplasm to face the challenges of the modern agriculture; landraces could be useful for this purpose.

## 5. Conclusion

This is the first report of the analysis of genetic relationships among a large sample of common bean advanced cultivars widely grown in North America using SSR markers. SSR markers used in the present study were appropriate to provide a first overview of the genetic diversity levels and of the population structure within the common bean cultivars.

The germplasm evaluation highlighted the existence of a broad genetic base in landraces and a narrowing of diversity in the advanced cultivars due to breeding activities. The investigation of population structure suggested the genetic potential of landraces for the detection of new sources of variation, and allowed us to identify groups of accessions differentiated at molecular level potentially useful in common bean breeding programs.

## Supporting information

S1 Table Seed list of 195 accessions used in this study, STRUCTURE membership coefficient for K = 2, and genotypic information (alleles) for each SSR locus. (XLSX)

S2 Table SSR markers used in this study. (XLSX)

S3 Table Seed list of 195 accessions used in this study and structure (K = 2, K = 3, K = 5 and K = 8) and DAPC membership coefficient (K = 7). (XLSX)

S4 Table Kwak and Gepts [7] dataset with DAPC membership coefficient. (XLSX)

S5 Table Membership coefficient of the common bean varieties on the genetic clusters identified by DAPC on the worldwide germplasm collection. (XLSX)

S6 Table 6a) Genetic differentiation based on *F*_*ST*_ (above the diagonal) and Nei Distances (below the diagonal) values between all pairwise combinations of common bean races. 6b) Genetic differentiation based on FST (above the diagonal) and Nei Distances (below the diagonal) values between all pairwise combinations of common bean market class.
(XLSX)

S1 Fig STRUCTURE estimation of the number of subpopulations for K ranging from 2 to 12 by mean likelihoods (A) and Delta K values (ΔK) (B).
(XLSX)
